# The Effect of Social Rank on Reproductive Traits Depends on Rank Metric: Evidence From a Group‐Living Carnivore

**DOI:** 10.1002/ece3.73229

**Published:** 2026-03-15

**Authors:** Ella W. White, Oliver P. Höner, Marta Mosna, Viktoriia Radchuk, Sarah Benhaiem, Eve Davidian

**Affiliations:** ^1^ Department of Evolutionary Ecology Leibniz Institute for Zoo and Wildlife Research Berlin Germany; ^2^ Department of Ecological Dynamics Leibniz Institute for Zoo and Wildlife Research Berlin Germany; ^3^ Department of Biology, Chemistry and Pharmacy Freie Universität Berlin Berlin Germany; ^4^ Evolutionary Anthropology Team, Institute of Evolutionary Science of Montpellier (ISEM) University of Montpellier, CNRS, IRD Montpellier France

**Keywords:** age at first reproduction, competitive context, interbirth interval, ordinal rank, reproductive success, spotted hyena, standardised rank

## Abstract

Group‐living animals form dominance hierarchies to mediate competition among members. The social rank of individuals determines their access to resources such as food and mates, as well as their exposure and opportunities to respond to social challenges. High rank often translates into greater reproductive success, but the strength of this correlation may vary considerably depending on the rank metric and the reproductive trait studied. Here, we investigated the predictive ability of two rank metrics—ordinal and standardised ranks—assumed to capture distinct underlying mechanisms of intragroup competition on six reproductive traits for female and male spotted hyenas *Crocuta crocuta* . We used 28 years of individual‐based data from 481 hyenas from the Ngorongoro Crater, Tanzania. Ordinal rank strongly outperformed standardised rank in its ability to predict four out of six traits. As ordinal rank reflects the absolute position of an individual in the hierarchy, its importance when predicting reproductive traits suggests the relevance of priority of access to resources for both sexes. One trait—interbirth interval—was instead best described by standardised rank, suggesting socially mediated constraints on reproductive output. Our results reveal mechanisms that have been previously overlooked in explaining rank‐related success in specific reproductive traits in the spotted hyena and emphasise the importance of selecting rank metrics that appropriately reflect the competitive context in focus.

## Graphical abstract

In animal societies, one's social rank determines access to resources like food, mates, and allies. Using longitudinal data on 481 spotted hyenas, we tested two common social rank metrics with different mechanistic assumptions to see whether resource access or social constraints could best explain different aspects of reproductive success. Our results emphasise how methodological choice—specifically how social rank is defined—shapes our understanding of what drives reproductive success in group‐living animals.

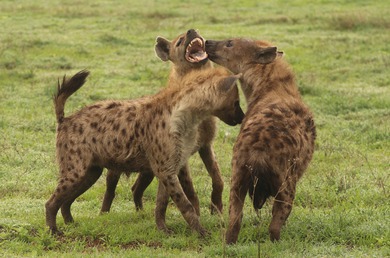

## 1 Introduction

In group‐living animals, competition for critical resources such as food or mating opportunities is often moderated by the formation of social dominance hierarchies (Tibbetts et al. 2022). An individual's position in a hierarchy strongly influences its access to these resources (Whitten 1983; Isbell et al. 1999), its social environment (Creel et al. 2013), and ultimately, its reproductive success (Ellis 1995; Stockley and Bro‐Jørgensen 2011; Majolo et al. 2012; Clutton‐Brock and Huchard 2013a; Shivani et al. 2022).

High‐ranking individuals usually experience priority access to food or mating opportunities, as social dominance allows for displacement of subordinate group members (Whitten 1983; Isbell et al. 1999). The resource in competition and intensity of intragroup competition may differ between females and males (Bateman 1948; Clutton‐Brock 2007). In mammals, female reproductive success is heavily constrained by energetics, and thus access to food, especially as gestation, lactation and provisioning of offspring increase energy demands (Gittleman and Thompson 1988; Speakman 2007; Emery Thompson 2017). Growth of offspring, and consequently their survival and age of maturity, is largely driven by maternal access to food (Maestripieri and Mateo 2009). Thus, in social structures with multiple breeding females (i.e., plural breeding groups), high‐ranking females often have an earlier age at first reproduction, shorter interbirth intervals, higher offspring survival, and greater overall lifetime reproductive success (Stockley and Bro‐Jørgensen 2011; Shivani et al. 2022). The intensity of competition and relative effect of rank on female reproductive success will additionally depend on the value and availability of food sources. Carnivorous and frugivorous species for example often rely on transient, patchy, high value foods, and this can lead to greater discrepancies in rank‐related reproductive success in females, effects of which can be further heightened or lost during periods of unnaturally low or high resource abundance (Clutton‐Brock and Huchard 2013a). Conversely, while competition for food involves all group members, male reproductive success is rarely determined by food access, which influences males only indirectly through body condition (Clutton‐Brock and Huchard 2013b). Instead, males are often limited by their relative access to mating opportunities, and thus compete intrasexually for courtship and copulation with receptive females (Clutton‐Brock 2007). High‐ranking males can be more successful in monopolising or ‘mate‐guarding’ females by displacing subordinate competitors, increasing individual reproductive success via successful paternities (Ellis 1995; Davidian et al. 2022). High‐ranking males may additionally improve their reproductive success by prioritising courtship of the highest quality females to ensure offspring survival and long‐term fitness payoffs (Setchell and Wickings 2006; Cafazzo et al. 2014; Davidian et al. 2016).

High‐ranking individuals often also benefit from a more favourable social environment than low‐ranking individuals. For example, enduring exposure to social challenges and agonism by low‐ranking individuals may induce heightened physiological costs (i.e., an elevated allostatic load) and impair fitness (Goymann and Wingfield 2004; Creel et al. 2013). High‐ranking individuals are often more integrated within the group social network and benefit from more grooming or coalitionary partners, which may otherwise help in relieving allostatic load (Abbott et al. 2003; Young et al. 2014; Canteloup et al. 2021). They also have more individuals below them in the hierarchy to redirect aggression toward as a coping mechanism (Sapolsky and Ray 1989; Abbott et al. 2003). The physiological demands of holding a certain rank can vary greatly between social systems and modes of rank establishment and maintenance (Creel 2001; Goymann and Wingfield 2004; Sapolsky 2005; Creel et al. 2013), and this may impact patterns of rank‐related reproductive success (Beehner and Lu 2013; Clutton‐Brock and Huchard 2013a). For example, in plural breeding societies where hierarchies are relatively stable over time and the positions of high‐ranking individuals rarely challenged, aggression may be expressed mostly down the hierarchy and low‐ranking individuals may bare higher allostatic load than their high‐ranking conspecifics (Goymann and Wingfield 2004; Sapolsky 2005; Davidian et al. 2021). Low‐ranking individuals may in turn adopt behavioural strategies to circumvent these physiological costs—typically by reducing their attendance at social hubs such as key feeding sites or dens—but this may incur nutritional, social, and ultimately reproductive costs (Briffa and Sneddon 2007; Marshall et al. 2012; Davidian et al. 2021).

Researchers have proposed a number of ways to infer dominance hierarchies from interaction data and calculate rank metrics (Sánchez‐Tójar et al. 2018). These metrics often reflect distinct mechanisms by which rank‐based competition operates and should affect a studied trait (Levy et al. 2020). One common way of determining the order of a hierarchy is achieved by assigning individuals linearly based on their ratio of wins/losses in agonistic dyadic interactions (de Vries 1998). This results in a simple ordinal rank, where the highest ranking individual is assigned ‘1’, and the lowest ‘*n*’, where *n* is the hierarchy length. This metric is often further modified to account for discrepancies in group size between different social groups and over time by scaling ordinal rank by hierarchy length (*n*), so that the highest ranking individual is assigned rank ‘1’, and the lowest ‘0’ or ‘−1’, depending on the study (i.e., ‘standardised rank’, also termed ‘relative’ or ‘proportional’ rank; Levy et al. 2020). Importantly, these two representations of the same hierarchy underlie different assumptions in how social rank reflects intragroup competition. Ordinal rank indicates the absolute position in the hierarchy, and thus the number of individuals that outrank the focal animal. This assumes that only the total number of competitors that rank higher than the focal animal are relevant to the competitive landscape, and that any ranking below, regardless of group size, will not change the intensity of rank‐based competition (Levy et al. 2020). Ordinal rank may thus better reflect competitive contexts that operate via priority of access. On the other hand, standardised rank reflects the ratio of conspecifics that are dominant versus those that are subordinate to the focal animal, assuming that the number of group members that are subordinate to the focal animal are as important as those that rank higher than them in influencing competitive mechanisms. Standardised rank therefore integrates availability of rank‐related stress coping outlets and opportunities to redirect agonism onto subordinate group members (Davidian et al. 2021). These biologically‐rooted, conceptual differences between ordinal and standardised ranks allow one to derive explicit predictions regarding their effects on specific fitness‐related traits depending on the study system. Perhaps most importantly, these metrics are obviously inequivalent, and thus use without considering assumptions of the expected mechanisms of competition can result in the failure to detect effects that a more appropriate metric may have found (Levy et al. 2020; Mielke 2023). It may further impair our ability to identify broad eco‐evolutionary patterns in comparative studies (Ellis 1995; Stockley and Bro‐Jørgensen 2011; Majolo et al. 2012; Clutton‐Brock and Huchard 2013a; Shivani et al. 2022). These issues have only been addressed recently and there is growing recognition that more empirical quantification is needed of how well different rank metrics reflect the mechanisms by which social rank influences fitness across species.

We aimed to determine which of ordinal or standardised rank best represents the relationship between dominance and six reproductive traits in both sexes of the spotted hyena *Crocuta crocuta* (hereafter hyenas). In doing so, we investigated whether these traits were better explained by mechanisms of intragroup resource competition (ordinal rank) or by physiological constraints driven by the social environment (standardised rank). For this, we conducted six analyses, four replicated from previously published analyses (Höner et al. 2010; Davidian et al. 2016; Gicquel et al. 2022) and two developed based on previously published nonparametric tests (Holekamp et al. 1996; East et al. 2003), using 28 years of individual‐based data from the Ngorongoro Crater, Tanzania. While competition underlying success in any trait is unlikely to be driven by a single mechanism, support for one metric over the other should help us to understand primary drivers of the trait considered. Recent pioneering work inspiring our study design suggests sex‐specific patterns relating to the density dependence of resources in baboon troops (Levy et al. 2020). The mechanisms underlying their predictions, however, do not translate well into the hyena social system. We instead predicted that each reproductive trait can be under a distinct competitive regime and will be best explained by the rank metric that best represents its underlying mechanism, irrespective of the sex of individuals. We thus outlined predictions for each trait. For female hyenas, reproductive traits should be most limited by energy, and therefore food access. As obligate carnivores, food is a transient and highly monopolisable resource, particularly for high‐ranking females, which not only have priority access to the carcass at kill sites, but also to preferred foraging grounds within the territory (Hofer and East 1993a, 2003; Smith et al. 2008). Territory size does not limit access to prey as hyenas regularly intrude into neighbouring clan territories when within‐territory prey abundance is low (Hofer and East 1993b; Höner et al. 2005). Hyenas additionally competently hunt solo or in small groups, and while hunting success is decoupled from group size, kills often attract other clan members to feed (Holekamp et al. 1997; Smith et al. 2008). We thus expected ordinal rank would best represent this access to energy—that is, the absolute ability to displace others at carcasses, regardless of group size—in female hyenas. Therefore, we expected the three reproductive traits for females—cub survival, interbirth interval and age at first reproduction—to be better predicted by ordinal rank than standardised rank (Table 1). For males, we predicted more nuanced results. Female hyenas exercise strong mate choice and cannot be sexually coerced, and so males must invest time and energy in courting and strengthening relationships with potential mates to increase chances of siring litters (Szykman et al. 2001; East et al. 2003; Davidian et al. 2021). Mating success is therefore improved by fostering these long‐term bonds with females, and so social integration and investment in courtship have important fitness consequences. Male ability to invest time and energy in courtship behaviours has been shown to correlate with male standardised rank and associated availability of stress coping outlets (Davidian et al. 2021). Thus, we predicted standardised rank to be a better predictor of annual reproductive success and age at first reproduction for males than ordinal rank (Table 1). Regarding mate quality, we expected that achieving mating success with a high‐ranking female (top‐quality mating partners) not only requires management of time invested in courtship, but the ability to exclude potential competitors. Yet, the ability to displace other males shadowing high‐ranking females should only be possible for the highest‐ranking suitors, regardless of the number of competitors below them in the hierarchy. We therefore predicted that ordinal rank should best describe the rank of mated female (Table 1).

**TABLE 1:** Summary of study predictions regarding whether ordinal or standardised rank best describe the relationship between social rank and six reproductive traits in spotted hyenas. Brief reference to underlying mechanisms discussed in the main text is included to support these predictions.

	Reproductive trait	Predicted best rank metric	Underlying mechanism
Females	Cub survival	Ordinal rank	Priority resource access to food
	Interbirth interval	Ordinal rank	Priority resource access to food
	Age at first reproduction	Ordinal rank	Priority resource access to food
Males	Annual reproductive success	Standardised rank	Courtship restraints imposed by social challenges
	Rank of mated female	Ordinal rank	Competitive exclusion of rival males
	Age at first reproduction	Standardised rank	Courtship restraints imposed by social challenges

## 2 Materials and Methods

### 2.1 Study Species

Hyena societies (termed clans) are comprised of overlapping generations of philopatric females, their natal offspring, and immigrant males. Clans vary greatly in size, from as few as three individuals to 130 (Vullioud et al. 2019). Individuals are considered cubs between 0 and < 12 months of age, and subadults between 12 and < 24 months. Males tend to disperse from their natal clan as adults between two and 5 years of age (on average, 3.5 years; Davidian et al. 2016) and join a new clan at the bottom of the hierarchy (termed ‘immigrant’ males). A smaller number of males begin their reproductive career in their natal clan, thus retaining their natal rank (termed ‘philopatric’ males). Clans are structured in strictly linear dominance hierarchies where each individual is ranked according to predictable social conventions, where cubs inherit the social rank directly below that of their mother and above all older maternal siblings (maternal rank inheritance and youngest ascendency; Holekamp and Smale 1991) and immigrant males generally ‘queue’ for social rank (East and Hofer 2001).

### 2.2 Field and Lab Methods

Data were collected from April 1996 to December 2024 as part of near‐daily monitoring of the Ngorongoro Crater (3°11′ S, 35°34′ E) spotted hyena population in Tanzania. All members of the eight clans that inhabit the Crater floor were individually known and identified by their unique spot pattern, ear notching, or scarring. Monitoring sessions typically occurred between 06:00 and 19:00 at communal dens, kill sites and common resting areas and involved noting observations of all individuals in the area, recording of behavioural information and opportunistic collection of samples. Cubs were sexed at approximately 3 months of age based on the appearance of the phallic glans and birthdates estimated with an accuracy of ±7 days based on pelage, size, and locomotion (Pournelle 1965; Frank et al. 1990). Date of conception was back‐calculated based on the known gestation period of 110 days (Matthews 1939).

To determine parentage, samples (e.g., faeces, tissue, hair) from known individuals were gathered opportunistically and non‐ or minimally invasively without captures or immobilisation. Samples were genotyped using nine highly polymorphic microsatellite loci following Wilhelm et al. (2003). Maternity and paternity were then determined via maximum likelihood methods implemented in CERVUS 3.0 (Kalinowski et al. 2007). See Höner et al. (2010) for further information.

### 2.3 Data Processing to Reduce Bias

Hyena pregnancies are difficult to detect visually and females give birth to their litters in isolated birthing dens, only transferring cubs to communal dens at several weeks of age (East et al. 1989). Even once moved to the communal den, young cubs will remain underground and may not be sighted unless the mother comes to nurse. Death of very young cubs may therefore lead to field observers ‘missing’ cubs or litters, particularly those of low‐ranking females, as they may transfer cubs to the communal den later and visit cubs less often (Hofer and East 1993c; Wachter et al. 2002). These ‘missed’ cubs are also particularly relevant for male reproductive traits, as paternity can only be confirmed via genetic assignment, and many young cubs may die before opportunistic faecal samples can be collected. To minimise potential bias in our analyses, we used data from the four clans (Airstrip, Lemala, Munge and Shamba) with highest observation effort over the full study period for reproductive traits that might be affected by ‘missing’ litters (i.e., cub survival, age at first reproduction, interbirth interval, annual reproductive success; Table 2, Table S1). Analyses of the rank of mated females were conducted on data from all eight study clans (as aforementioned, plus Engitati, Forest, Ngoitokitok, and Triangle). Extremely rare cases of extraclan matings were excluded from all analyses (Davidian et al. 2016).

**TABLE 2:** Reproductive traits investigated in this study as a function of two social rank metrics (ordinal/standardised) and other predictors. Each model structure was fitted two times: one where rank and associated variables (Δrank and rank^2^) were ordinal and the other was standardised. The ‘:’ indicates an interaction effect. See Table S1 for all variable definitions and justifications.

Sex	Trait (response variable)	Model type	Model structure (predictor variables)	Original model citation	Sample sizes
Female	Cub survival	GLMM (binomial, logit link)	rank + Δrank + litter status + sex + age + number of lactating females + (1|ID) + (1|year)	Gicquel et al. (2022)^a^	1367 cubs 274 females
	Interbirth interval	LMM	rank + rank^2^ + Δrank + age + litter size + (1|ID)	N/A^b^	535 intervals 202 females
	Age at first reproduction	LMM	rank + rank^2^ + Δrank + litter status + maternal age + number of lactating females + (1|mother ID)	Gicquel et al. (2022)^c^	243 females
Male	Annual reproductive success	GLMM (negative binomial (nbinom2), log link)	rank + Δrank + male origin + number of young females + maternal rank + year of tenure + male origin: year of tenure + (1|ID)	Davidian et al. (2016)	933 tenure years 773 cubs 194 males
	Rank of mated female	LMM	rank + male origin + maternal rank + year of tenure + male origin: year of tenure + (1|ID)	N/A^b^	625 conceptions 233 females 132 males
	Age at first reproduction	LM	rank + Δrank + number of young females + litter status + clan	Höner et al. (2010)	137 males

^a^ Cub survival in Gicquel et al. (2022) was tested using a multivariate generalised linear model and thus our model is not an exact replicate. Note that this study also focused on a different population of hyenas in the Serengeti and restricted analyses of survival to female cubs.

^b^ Note that while interbirth interval and rank of mated female have been published in studies, tests were non‐parametric and so no model structure could be replicated (Holekamp et al. 1996; East et al. 2003). Model structure for these traits was chosen a priori based on covariates known to influence similar traits.

^c^ Age at first reproduction in Gicquel et al. (2022) was tested using a multivariate Hazard‐Cox model and thus our model is not an exact replicate. Note that this study also modelled a different population of hyenas in the Serengeti.

### 2.4 Social Rank Assignment

Ordinal rank was initially derived from a sociometric matrix constructed for each clan once 80% of dyadic interaction outcomes between members had been observed at the beginning of the long‐term research project in April 1996. Ranks were assigned linearly—the individual with the highest proportion of wins assigned as ordinal rank 1. This matrix was then continuously updated upon demographic events such as births, deaths and immigration with new individuals assigned a rank according to aforementioned predictable social conventions. Re‐ordering occurred iteratively when this rank based on social conventions contradicted new interaction outcomes, suggesting rank reversals (for additional information, see Davidian et al. 2021). Ordinal rank on a certain date was then extracted based on this matrix. We obtained standardised ranks by scaling ordinal ranks by hierarchy length from −1 (lowest ranking) to 1 (highest ranking), consistent with previous literature on hyenas seeking to account for differences in clan size and/or coping opportunities (e.g., East and Hofer 2001; Davidian et al. 2021; Gicquel et al. 2022).

When determining social rank for females, we built the hierarchy from all clan members who were considered subadult or older (≥ 12 months old). Importantly, subadult and native adult males (sexually inactive or philopatric) retain their maternally‐inherited social rank, and thus can be higher ranked than some females. This gives them the ability to dominate and displace lower‐ranking females at kills, and so such males are valid competitors and sources of aggression (Vullioud et al. 2019). Cubs (< 12 months old) were excluded as social rank takes 6–18 months to ‘learn’ and during this period, cubs are not yet consistent aggressors nor victims in social situations (Smale et al. 1993; Strauss et al. 2020). Finally, inclusion of immigrant males represents more opportunity for females to direct or redirect aggression.

For males, sexual activities and reproductive success should be influenced by the ability to displace other males and mate guard females, which only stems from intrasexual competition. For determining social rank for males, we therefore only included sexually active males in the hierarchy—that is, philopatric and immigrant males. As philopatric males are far less common and the majority of males rank below all females in the clan, there is less need to quantify the number of females above them, as all immigrant males will be regular targets of dominant behaviours by females. A male is only considered to have chosen a clan and join its hierarchy when he both expressed sexual behaviour toward females and began to invest socially in other sexually active males in the clan (Davidian et al. 2016).

### 2.5 Statistical Methods

The reproductive traits and their predictor variables, described in full in Table S1, were taken directly from previous models in the literature or chosen a priori based on known influence on reproductive success (Holekamp et al. 1996; East et al. 2003; Höner et al. 2010; Davidian et al. 2016; Gicquel et al. 2022). Where possible, variables were standardised across models. Individual social rank was determined on a given date relevant to the trait of interest. As many reproductive traits are not well captured by a social rank on a single date, we also included the variable Δrank to control for the magnitude of change in social rank over the period considered for these traits. The majority of changes in social rank are minimal as hyena hierarchies are relatively stable over time. However, sudden rearrangements of the hierarchy (‘coups’) occur infrequently, leading to larger rank differences for which Δrank helps control for. This was calculated as the difference in ordinal or standardised rank between the start and end of the considered period (see Table S1 for descriptions of considered periods for each trait). We calculated both individual ordinal and standardised rank at each of these instances for comparison.

We built two identical models to explain each trait—one for ordinal rank and one for standardised rank (Table 2). Where possible, models were replicated from previous publications using the Ngorongoro Crater population but utilising an updated dataset (Höner et al. 2010; Davidian et al. 2016) or based on previous publications using other hyena populations (Gicquel et al. 2022). Model structures for relationships between interbirth interval and rank and rank of mated female and rank tested in the past with non‐parametric tests were built a priori with covariates known to influence similar traits (Holekamp et al. 1996; East et al. 2003).

To meet model assumptions regarding residual patterns and general goodness‐of‐fit, we inverse square root transformed interbirth interval, log transformed age at first reproduction for both sexes, and square root transformed rank of mated female. Additionally, interbirth interval and age at first reproduction required inclusion of a quadratic effect of rank as a predictor in order to satisfy model assumptions (Table 2). We removed several data points (maximum removed was 3% in analyses on female age at first reproduction) that were either biologically non‐meaningful or where data were very sparse, causing problems with model diagnostics (especially at low ranks; see Table S2 for case‐specific justification).

To compare paired models, we used the difference in Akaike's information criterion corrected for small sample sizes (ΔAICc; Burnham and Anderson 2004). A difference of < 2 ΔAICc implies that there is no evidence to discriminate between the two models based on the data. ΔAICc of 2–7 implies a tendency toward data support for one model over the other, but still some support for both formulations. ΔAICc > 7 implies stronger data support for one model over the other (Burnham et al. 2011). To ensure that the inclusion of rank explained variation in the data above that explained by the covariates, we also calculated the AICc for a ‘null’ model that did not include ordinal rank, standardised rank, quadratic effects, and Δrank where applicable, and compared this ‘null’ model to the models with rank‐related variables included. We defined the best performing model as the model with a difference of at least 2 AICc less than the next best model.

All analyses were performed in R version 4.3.1 (R Core Team 2023). Data extraction, filtering and creation of clean datasets used the Ngorongoro Hyena Project package *hyenaR* 0.10.0 (Bailey et al. 2024). Linear models (LMs) were performed with base R. Linear mixed models (LMMs) and generalised linear mixed models (GLMMs) were performed with the package *glmmTMB* version 1.1.9 (Brooks et al. 2017). Model diagnostics were assessed using package *DHARMa* version 0.4.6 (Hartig 2024). Figures were produced using packages *ggplot* version 3.5.1 (Wickham 2016), *ggeffects* version 1.6.0 (Lüdecke 2018) and *patchwork* version 1.3.0 (Pedersen 2025).

## 3 Results

**FIGURE 1:**
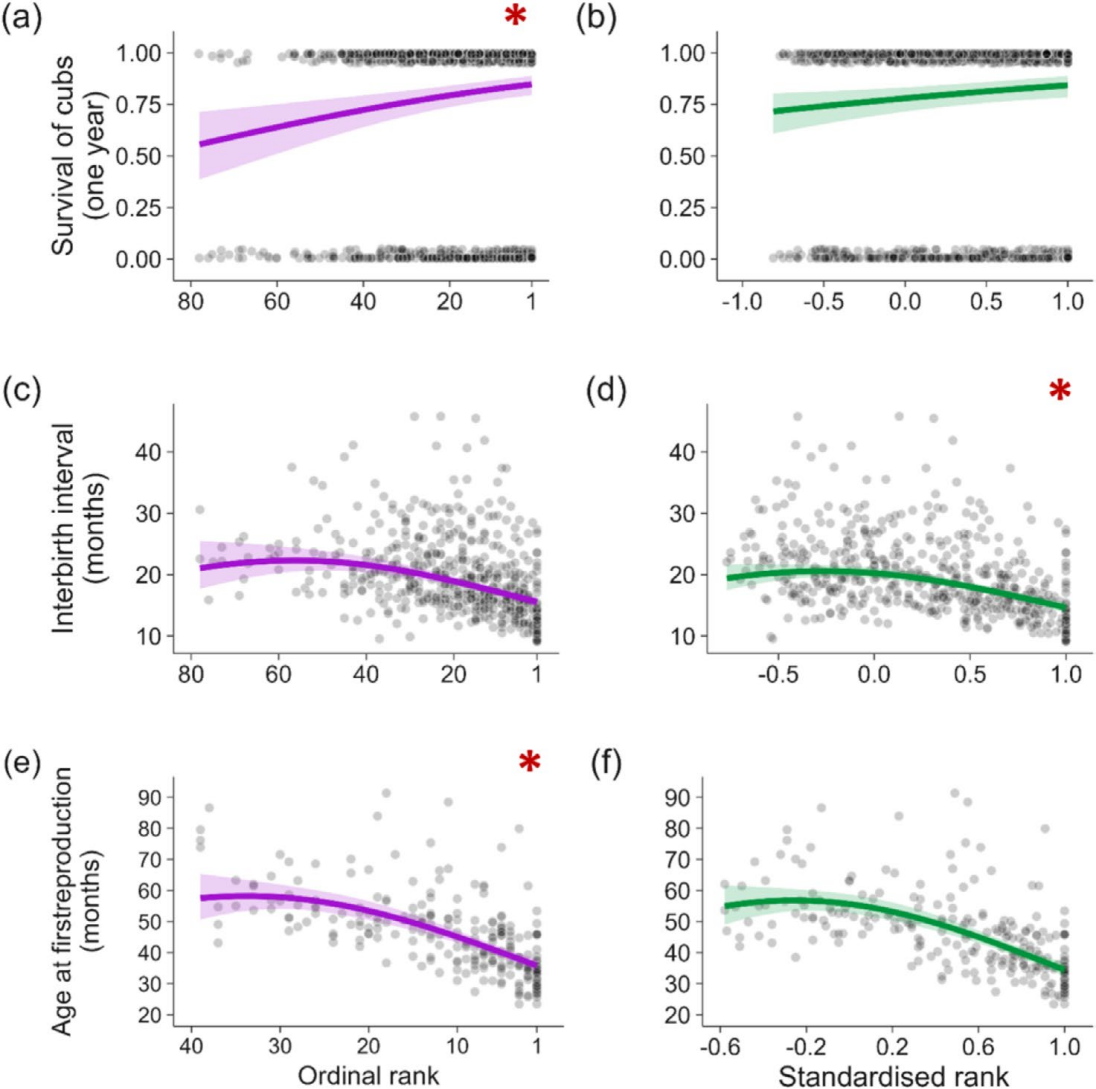
Relationship between female reproductive traits and ordinal rank (purple; a, c, e) and standardised rank (green; b, d, f). (a, b) Probability of cub survival to 1 year. (c, d) Duration of the interbirth interval in months following a successful litter. (e, f) Age (months) at which a female gave birth to her first litter. Larger numbers in (a, c, e) represent a lower ordinal rank, while larger numbers in (b, d, f) represent a higher standardised rank; Rank 1 corresponds to the top‐ranking individual in the hierarchy for both metrics. Transformed variables (interbirth interval, age at first reproduction) were backtransformed for visualisation. Red asterisks denote the preferred model of the pair determined by ΔAICc. Note differences in *x*‐axis scales within rank metrics. Points represent raw data and were jittered to avoid major overlap of points. Solid lines represent the effect size and ribbons represent the 95% confidence intervals as predicted when all other covariates were held constant by R package ggeffects (see Supporting Information S3 for constant values).

**FIGURE 2:**
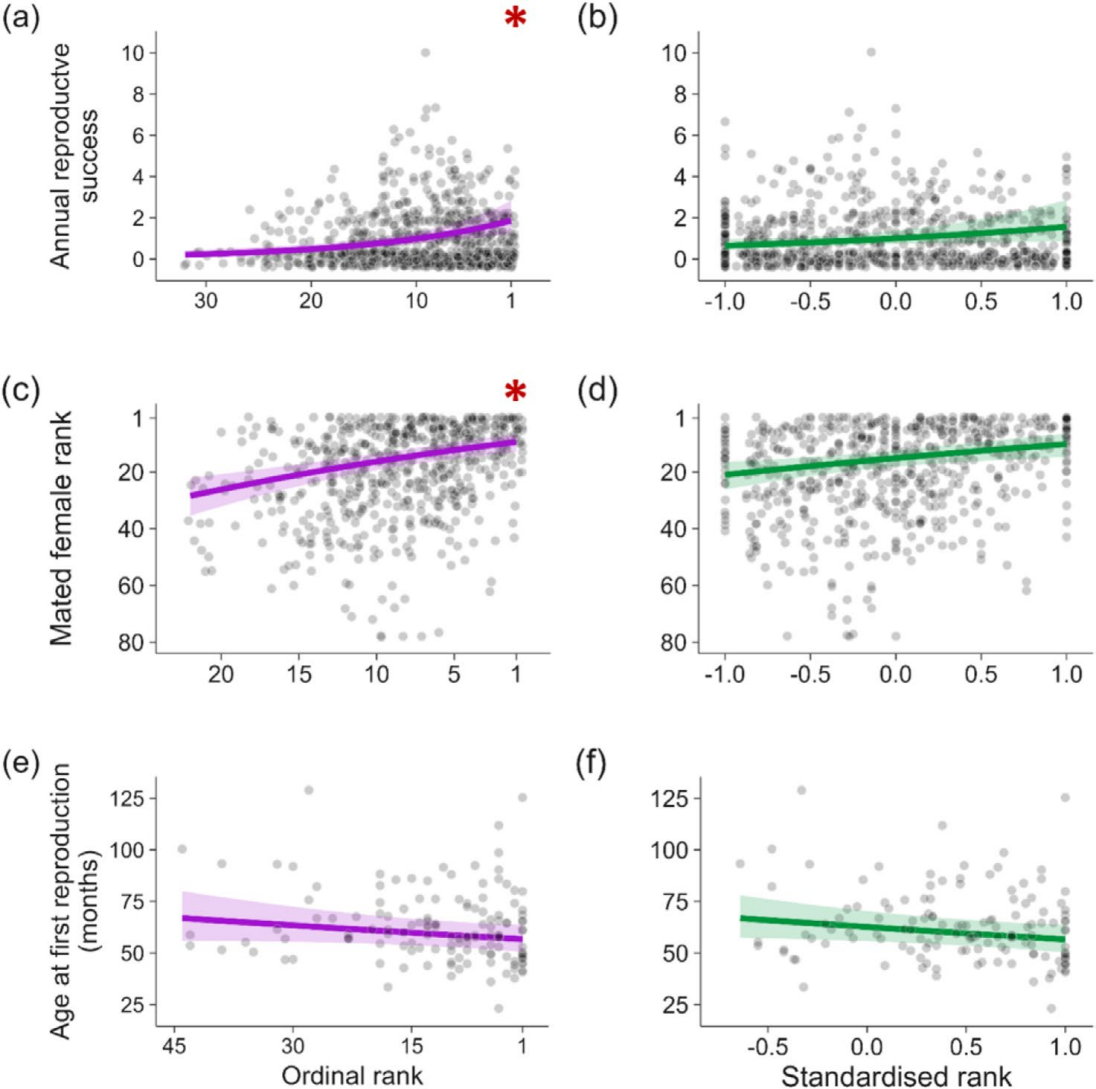
Relationship between three male reproductive traits and ordinal rank (purple; a, c, e) and standardised rank (green; b, d, f). (a, b) Annual number of cubs sired by a male. (c, d) Rank of a female confirmed to have been mated with (i.e., who gave birth to cubs with paternity assigned to the focal male) on the estimated date of litter conception. (e, f) Age in months at which a male was at the estimated date of his first litter conception confirmed via paternity analysis. Larger numbers in (a, c, e) represent a lower ordinal rank, while larger numbers in (b, d, f) represent a higher standardised rank; Rank 1 corresponds to the top‐ranking individual in the hierarchy for both metrics. Transformed variables (Rank of mated female, age at first reproduction) were backtransformed for visualisation. Red asterisks denote the preferred model of the pair determined by ΔAICc. For age at first reproduction, neither model fit the data particularly better (see results). Note differences in *x*‐axis scales within rank metrics. Points represent raw data and were jittered to avoid major overlap of points. Solid lines represent the effect size and ribbons represent the 95% confidence interval as predicted when all other covariates were held constant by R package ggeffects (see Supporting Information S3 for constant values).

The reproductive success of female and male hyenas increased with social rank in all traits regardless of the metric used (Figures 1 and 2, Tables S3–S20). For five out of the six traits, both rank metrics better predicted the relationship than the null model (i.e., outperformed the null model by at least a ΔAICc of 2; Table 3) and the better performing model always held substantially more support from the data than the null model (ΔAICc ≥ 7). The exception was male age at first reproduction, where neither rank metric model performed better than the null model (ΔAICc ≤ 2).

**TABLE 3:** Ability of two rank metrics to explain reproductive traits in the spotted hyena. Sex and trait refer to the reproductive trait modelled. Rank metric refers to whether the model included ordinal rank, standardised rank, or an absence of rank metric as a predictor. The null model indicates the absence of not only the rank predictor variable but also corresponding quadratic terms and Δrank where applicable. AICc = the Akaike's information criterion corrected for small sample sizes, df = degrees of freedom. ΔAICc = the difference in AICc between the model considered and the best model, in bold. For male age at first reproduction, all models were equivalent; there was no best model.

Sex	Trait	Rank metric	df	AICc	ΔAICc
Female	Cub survival	**Ordinal**	**10**	**1524.671**	**0**
		Standardised	10	1535.41	10.74
		Null model	8	1538.57	13.90
	Interbirth interval	**Standardised**	**8**	**−2266.50**	**0**
		Ordinal	8	−2243.26	23.25
		Null model	5	−2198.74	67.77
	Age at first reproduction	**Ordinal**	**10**	**−108.38**	**0**
		Standardised	10	−97.21	11.18
		Null model	7	−12.26	96.12
Male	Annual reproductive success	**Ordinal**	**20**	**2287.03**	**0**
		Standardised	20	2305.65	18.62
		Null model	18	2308.14	21.11
	Rank of mated female	**Ordinal**	**19**	**2431.10**	**0**
		Standardised	19	2447.91	16.81
		Null model	18	2454.28	23.18
	Age at first reproduction	Standardised	9	39.10	0
		Null model	7	39.77	0.67
		Ordinal	9	40.02	0.92

All traits had strong support for one rank metric over the other (ΔAICc ≥ 7; Table 2), with the exception of male age at first reproduction, for which the difference between models was negligible (ΔAICc = 0.92; Table 3). Cub survival, female age at first reproduction, male annual reproductive success and rank of mated female all had greater support for the model using ordinal rank over standardised rank (Table 3). Interbirth interval of females was better predicted by standardised rank, and was the reproductive trait with the greatest difference in AICc between ordinal and standardised rank (ΔAICc = 23.2; Table 3). Thus, three of the six tested traits (cub survival, female age at first reproduction, and rank of mated female) favoured the rank metric outlined in our predictions, that is, that ordinal rank better explains reproductive traits that are driven by priority access to key resources via exertion of high social rank.

## 4 Discussion

Using long‐term data on female and male spotted hyenas in the Ngorongoro Crater, Tanzania, we compared the predictive ability of two widely used social rank metrics on six reproductive traits. Inclusion of ordinal or standardised rank contributed substantially to explaining variability in all reproductive traits, except age at first reproduction in males. All traits followed trends where a higher rank resulted in a greater reproductive success. As expected, no metric prevailed for all traits, although ordinal rank did outperform standardised rank in most (four out of six) traits, suggesting the general importance of priority of resource access that comes with holding high rank for improving reproductive success. Of the six predictions regarding which rank metric and therefore which competitive mechanism—that is, priority of resource access or socially mediated physiological constraints—should best predict the reproductive traits (Table 1), three were confirmed. We discuss these individually, outline how each reproductive trait may be mediated by which competitive mechanism, and how our results contribute to an emerging framework of trait‐specific, inference‐based choice of rank metric.

Reproductive success in female mammals is constrained by the energetic demands of gestation, lactation and maternal care. As priority access to food is so crucial for female hyenas, we expected—and confirmed—that the rank metric reflecting this mechanism of competition, ordinal rank, better described the relationship between female social rank and both cub survival and age at first reproduction, two traits that contribute greatly to overall lifetime reproductive fitness in long‐lived mammals (Martin and Festa‐Bianchet 2012; Hayward et al. 2014). Female mammals with access to food in greater amounts and/or of higher nutritional value produce milk with higher protein and fat content and in larger volumes (Del Prado et al. 1997; Landete‐Castillejos et al. 2003). The quality and quantity of maternal milk directly affect the growth of their offspring, and the body mass of juveniles correlates with survival (Ronget et al. 2018). Additionally, the timing of sexual maturity in mammals is tightly linked to early‐life nutrition and speed of growth. This has been shown both experimentally in captive rhesus macaques *Macaca mulata* (Terasawa et al. 2012) and field studies of European badgers *Meles meles* (Sugianto et al. 2019). Similarly, in spotted hyenas, females that grow faster early in life—due to their mother's high social rank and better access to resources—tend to survive better to adulthood and reproduce earlier (Hofer and East 2003).

We predicted interbirth interval, driven strongly by the duration of previous offspring dependence and the ability of the mother to recover from the costs of lactation, to be influenced by the same underlying mechanisms as cub survival and female age at first reproduction. Standardised rank, however, strongly outperformed ordinal rank for interbirth interval. Resource access is likely still an important driver, but its effect on interbirth interval may be mediated by the ability of mothers to cope with social challenges when attending the den to nurse their cubs. Hyena cubs are kept at communal dens, which also act as a social hub for the clan where individuals of all social rank meet, interact, and foster bonds (White 2007; Strauss et al. 2024). Attending these sites is key to maintaining integration in the clan social network. Communal dens are therefore the only location in clan territories (aside from kill sites) where agonism from higher‐ranking individuals may not be easily avoided. Low‐ranking hyena mothers are also more likely to be interrupted by higher‐ranking individuals mid‐nursing to elicit submission and denied access to the den entrance by higher‐ranking individuals (East et al. 1989; White 2007). Denial of access to offspring may reduce nursing frequency, and forceful interruption mid‐bout may lead to insufficient transfer of milk per bout. Lactation is often the most physiologically costly phase of reproduction, leading to higher allostatic load independent of social rank (Gittleman and Thompson 1988; Goymann et al. 2001; Speakman 2007). While this leads to the clear rank‐based effect on allostatic load in female hyenas being equalised during lactation (Goymann et al. 2001), the fact that low‐ranking females must sustain an elevated allostatic load for a longer period of time due to slower cub growth may additionally require a longer recovery period before being able to conceive another litter. Low‐ranking females may additionally adopt suboptimal behavioural strategies to lessen their exposure to social challenges, for example by reducing their visits to the den and shifting their visits to times when temperatures are warmer and the risk of anthropogenic disturbances is increased (personal obs.). Similar trade‐offs between maternal care and avoidance of aggression from conspecifics have been documented in brown bears *Ursus arctos* , where mothers with cubs move into dangerous anthropogenic habitats to actively avoid conflict with dominant males (Elfström et al. 2014; de Van Walle et al. 2019). Although success in terms of offspring survival and maturation are mediated by resource access, mothers themselves may be more strongly limited in the frequency they can reproduce by physiological constraints resulting from compromises they must make to cope with their social environment.

As the unique reproductive anatomy of female hyenas gives them high reproductive control, male hyenas cannot sexually coerce potential mates (East et al. 2003; Szykman et al. 2007). Males instead increase their chances to be chosen by females as potential sires, and therefore their reproductive success, by investing in long‐term affiliative bonds and courtship activity. Early studies suggested that when females were courted by multiple males, the highest ranking of these potential mates was no more likely to succeed in siring her cubs than any other male she was observed with (Engh 2002; Szykman et al. 2007). Further, there appeared to be no correlation between the standardised social rank of males and that of the female they successfully sired cubs with (East et al. 2003). Here, we found that both annual reproductive success and the rank of mated female for males were better explained by ordinal rank—suggesting the importance of priority access to resources. Our results indicate that the absolute position in the male hierarchy clearly gives male hyenas reproductive advantages in both quality and quantity of offspring, suggesting potential benefits of competitive exclusion. As adult social rank is queued for (or inherited, in case of philopatric males), the achievement of more paternities and paternities of higher quality cubs is unlikely to be a result of superior sperm quality or any other intrinsic trait. Much of the literature on hyenas has focussed on female mate choice due to the high reproductive control that they hold in this society. Early studies suggested that male courtship behaviour and mate guarding do not improve chances of siring cubs (East et al. 2003; Szykman et al. 2007). However, the ability to court a targeted, high‐quality female without potential interruption or displacement—more likely for high‐ranking males—may be just as beneficial as active and targeted competitive exclusion in gaining more successful copulations in social species, and is rarely discussed as a significant benefit of high rank in male hyenas.

Our results for age at first reproduction for males did not find support for one rank metric over the other, and neither metric sufficiently outperformed the null model. While previous research suggested a strong effect of standardised maternal rank on age at first reproduction (Höner et al. 2010), it is reasonable that of those we tested, this trait had the weakest relationship with social rank. Male cubs of high‐ranking females, like female cubs, benefit from faster somatic growth due to a higher quality of maternal care, but there is currently no evidence that this leads to more rapid sexual maturity or an earlier age at dispersal (Höner et al. 2010; Davidian et al. 2016). Previous studies revealed that post‐dispersal males born to high‐ranking mothers regularly return to their natal territory after having dispersed to a new clan and benefit from enduring privileged access to food and social coping mechanisms (Höner et al. 2010). Our lack of evidence regarding the effect of rank on this trait may in part be due to the more complex and potentially contrasting mechanisms that underlie when males are first successful at siring cubs. The physiological and energetic costs associated with dispersal and integration into a new hierarchy (Davidian et al. 2021) may weaken the effect of a high maternal rank and its enduring benefits. Physiological and energetic costs of prospecting and dispersal are documented in social species such as meerkats *Suricata suricatta* (Young and Monfort 2009; Maag et al. 2019) and chimpanzees *Pan troglodytes* (Kahlenberg et al. 2008). Individuals may alleviate costs of dispersal by dispersing in pairs or groups (Schoof et al. 2009) (e.g., meerkats—Harrison et al. 2021, vervet monkeys *Chlorocebus pygerythrus* —L'Allier et al. 2022). In our study population, males preferentially coordinate dispersal alongside their twin brothers or close kin, particularly when clan density is high (Davidian and Höner 2022). It may well be that, as population size has increased over the study period, males born to low‐ranking mothers are just as likely to disperse with kin, and this may offset the benefit of high standardised maternal rank previously recorded in Ngorongoro hyenas (Höner et al. 2010; Davidian et al. 2016). However, further investigation is needed to understand under what conditions post‐dispersal males benefit from being born to high‐ranking mothers.

Our analyses join those of Levy et al. (2020) in underscoring that rank metrics cannot be treated as interchangeable descriptors of hierarchy position. As research‐derived concepts, rank metrics represent distinct hypotheses about the nature of processes shaping variation in traits. Selecting which rank metric to use is therefore not a trivial methodological decision, but a statement of which mechanistic assumptions are being made regarding competitive and social contexts. Additionally, our results show that testing multiple rank metrics with different underlying assumptions can provide a powerful framework for inferring what primarily shapes variation in individual traits, and can be used to highlight alternative explanations when results do not match predictions. While our evidence and that of others come from group‐living mammals that form strict linear dominance hierarchies (primates—Levy et al. 2020; Mielke 2023—and hyenas), these general findings are likely to be applicable across taxa and diverse social systems, taking care to consider species‐ and trait‐specific assumptions. It is important to reiterate that our results rely on assumptions based on expert knowledge of the competitive landscape that each rank metric approximates in hyenas, and our study does not directly measure the extent of resource access nor the allostatic load of individuals. While we took measures to mitigate potential observational bias present in non‐invasive data collection, an enduring limitation of studying male reproductive success in particular is the reliance on paternity data, which requires sired offspring to survive long enough for a sample to be collected and sequenced. However, since cub survival is driven strongly by maternal rank, and we show clear evidence of high‐ranking males achieving more paternities with high‐ranking females, we are confident that observed patterns are not heavily influenced by this limitation. We additionally do not anticipate that having restricted some analyses to only four clans limits our conclusions both within and beyond the Ngorongoro Crater population. A key benefit of such longitudinal data is that temporal variation within clans is as great as or even greater than variation between clans regarding hierarchy size, stability, and demographic structure.

Our study joins a growing body of literature highlighting that social rank metric choice influences the interpretation of studies on the ecology and evolution of group‐living animals (Levy et al. 2020; Mielke 2023). The conclusion of such evidence should not be to blindly test rank metrics to find the ‘best fit’ for a specific system, but instead curate study design and specifically use rank metrics that best represent the underlying mechanisms suspected to underlie hypotheses and predictions. Rank metric choice may also be used to explain unexpected results and suggest alternative mechanisms—acknowledging that interpretations may differ if approaching a question from an alternative starting point (Mielke 2023). The exception is perhaps when using social rank as a controlling variable in analyses exploring questions separate from social processes in which case, selecting a metric that best describes the effect that social rank has on the explanatory variable, regardless of mechanism, may be preferred. We additionally highlight in this study important aspects of the mechanisms underlying hyena reproductive biology. In females, interbirth interval—a critical trait for long‐term reproductive success in long‐lived animals such as the spotted hyena—may be more affected by social challenges than energetic restrictions. In males, the role of competitive exclusion in influencing female mate choice should be more strongly considered as a benefit of high social rank. Our results underline that biologically grounded methodological choice in behavioural ecology may be as powerful as data itself in explaining individual variation.

## Author Contributions

**Ella W. White:** conceptualization (equal), formal analysis (lead), methodology (equal), validation (lead), visualization (lead), writing – original draft (lead), writing – review and editing (lead). **Oliver P. Höner:** conceptualization (equal), data curation (equal), funding acquisition (equal), investigation (equal), methodology (equal), project administration (equal), supervision (equal), writing – review and editing (equal). **Marta Mosna:** investigation (equal), methodology (supporting), validation (equal), writing – review and editing (equal). **Viktoriia Radchuk:** conceptualization (equal), funding acquisition (equal), methodology (equal), supervision (equal), writing – review and editing (equal). **Sarah Benhaiem:** conceptualization (equal), funding acquisition (equal), methodology (equal), supervision (equal), writing – review and editing (equal). **Eve Davidian:** conceptualization (equal), data curation (equal), investigation (equal), methodology (equal), project administration (equal), supervision (equal), writing – review and editing (equal).

## Funding

This work was supported by the Leibniz Institute for Zoo and Wildlife Research Berlin and the German Federal Ministry of Research, Technology and Space (Bundesministerium für Forschung, Technologie und Raumfahrt; BMFTR) as part of the WILDER project (16DKWN148).

## Conflicts of Interest

The authors declare no conflicts of interest.

## Supporting information


**Data S1:** ece373229‐sup‐0001‐supinfo.docx.

## Data Availability

The data that support the findings of this study are openly available in figshare at https://doi.org/10.6084/m9.figshare.30327694.
