# Supplementary material for: The Effect of Social Rank on Reproductive Traits Depends on Rank Metric: Evidence From a Group‐Living Carnivore
Source: Ecol Evol. 2026 Mar 15;16(3):e73229. doi: 10.1002/ece3.73229 (PMC13093356; doi:10.1002/ece3.73229)
Supplement: Supplementary file 1 — Data S1: ece373229‐sup‐0001‐supinfo.docx. [file ECE3-16-e73229-s001.docx]

**Supplementary Information**

The effect of social rank on reproductive traits depends on rank metric: Evidence from a group-living carnivore

Ella W. White^1,2,3*^, Oliver P. Höner^1^, Marta Mosna^1,3^, Viktoriia Radchuk^2^, Sarah Benhaiem^2^, Eve Davidian^4^

^1^Department of Evolutionary Ecology, Leibniz Institute for Zoo and Wildlife Research, Berlin, Germany

^2^Department of Ecological Dynamics, Leibniz Institute for Zoo and Wildlife Research, Berlin, Germany

^3^Department of Biology, Chemistry and Pharmacy, Freie Universität Berlin, Berlin, Germany

^4^Evolutionary Anthropology Team, Institute of Evolutionary Science of Montpellier (ISEM), University of Montpellier, CNRS, IRD, Montpellier, France

* Corresponding author: white@izw-berlin.de

**This document is structured as follows:**

[**S1. Supplementary methods tables** 3](#_Toc210832604)

[Table S1. Model variables used across models and their definitions as applied to datasets on female and male spotted hyenas. 3](#_Toc210832605)

[Table S2. Number and characteristics of outliers/sparse data and their justification for removal from each dataset used 6](#_Toc210832606)

[**S2. Supplementary result tables: model outputs** 7](#_Toc210832607)

[Table S3. Model output for female cub survival (ordinal rank). 7](#_Toc210832608)

[Table S4. Model output for female cub survival (standardised rank) 7](#_Toc210832609)

[Table S5. Model output for female cub survival (null model) 8](#_Toc210832610)

[Table S6. Model output for female interbirth interval (ordinal rank). 8](#_Toc210832611)

[Table S7. Model output for female interbirth interval (standardised rank) 9](#_Toc210832612)

[Table S8. Model output for female interbirth interval (null model) 9](#_Toc210832613)

[Table S9. Model output for female age at first reproduction (ordinal rank) 10](#_Toc210832614)

[Table S10. Model output for female age at first reproduction (standardised rank) 10](#_Toc210832615)

[Table S11. Model output for female age at first reproduction (null model) 11](#_Toc210832616)

[Table S12. Model output for male annual reproductive success (ordinal rank) 11](#_Toc210832617)

[Table S13. Model output for male annual reproductive success (standardised rank) 12](#_Toc210832618)

[Table S14. Model output for male annual reproductive success (null model) 13](#_Toc210832619)

[Table S15. Model output for male mate rank (ordinal rank) 14](#_Toc210832620)

[Table S16. Model output for male mate rank (standardised rank) 15](#_Toc210832621)

[Table S17. Model output for male mate rank (null model) 16](#_Toc210832622)

[Table S18. Model output for male age at first reproduction (ordinal rank) 16](#_Toc210832623)

[Table S19. Model output for male age at first reproduction (standardised rank) 17](#_Toc210832624)

[Table S20. Model output for male age at first reproduction (Null model) 17](#_Toc210832625)

[**S3. Supplementary results: constant covariate values for marginal effects** 18](#_Toc210832626)

**Supplementary references**……………………………………………………………………………………………………………….19

#

# **S1. Supplementary methods tables**

Table S1. Model variables used across models and their definitions as applied to datasets on female and male spotted hyenas.

|  | Variable | Definition |
| --- | --- | --- |
| *Response* | Cub survival (females) | Binary variable (1/0) representing whether or not a (female or male) cub survived until one year after their date of birth. Social rank was determined on the birthdate of the cub. |
|  | Interbirth interval (females) | Continuous variable representing the time in months after a successful litter until another litter was born. A successful litter was defined as at least one cub surviving 12 months. In primatology, interbirth interval typically is calculated as the duration between two live births [1]. Applying such definition to hyenas may not accurately reflect the mother’s recovery from the combination of gestation, lactation and cub rearing because female hyenas may become receptive between three weeks and two months after losing a litter (in captivity; [2]). Social rank was determined on the birthdate of the cub/litter born at the beginning of the interbirth interval. |
|  | Age at first reproduction (females, males) | Continuous variable representing the age in months of a hyena on the date of first parturition (females) or on the estimated date of first conception (males). We used the social rank of the mother, i.e., ‘maternal rank’ of the focal individual because maternal rank is inherited by the individual, and it further reflects the quality of the early maternal environment (see [3,4]). Maternal rank was determined on the day of the focal individual’s birthdate. |
|  | Annual reproductive success (males) | Continuous variable representing the number of cubs conceived per year of tenure for each reproductively active male. Years were calculated as 12-month periods starting on the date on which a male chose its breeding clan. Only complete years of tenure were considered – i.e., when a male died or dispersed before 12 months had passed, this year was excluded from the analyses. Social rank was determined on the first day of that year of tenure (see [5]). |
|  | Mate rank (males) | Integer variable representing the ordinal rank of the mated female on the date of conception for each litter confirmed to have been sired via genetic testing. In cases of rare multiple paternity litters, the female was counted for both confirmed sires. Social rank of the focal male and the ordinal rank of the mated female were determined on the estimated date of litter conception. |
| *Fixed* | Ordinal rank | Integer variable representing the absolute position of an individual in the dominance hierarchy of their clan on a given date. An ordinal rank of 1 therefore represents the alpha position, and *n* (where *n* is the clan size) the lowest ranking position in the hierarchy. See *2.1.3. Social rank calculation* in main text for more information regarding determination of ordinal rank. To compare directionality of effects that different rank metrics have on reproductive traits, we inverted the ordinal rank, so that the highest-ranking individual was -1, and the lowest -*n*. Thus, both rank metrics had larger numbers representing higher rank. For the sake of visualisation, ordinal rank on the x-axis in figures was reverted to positive numbers. |
|  | Standardised rank | Continuous variable representing the proportional rank of an individual in their clan on a given date, obtained by standardising the ordinal rank to account for hierarchy size. A standardised rank of 1 therefore represents the alpha position, and -1 the lowest ranking position in the hierarchy. See *2.1.3. Social rank calculation* in main text for more information regarding determination of standardised rank. |
|  | Δrank | Integer or continuous variable representing the difference in social rank (ordinal or standardised) between two dates to control for substantial changes in rank over the period considered. For reproductive traits mediated by the early life environment (cub survival, age at first reproduction), Δrank was calculated as the difference in rank between date of birth and date at one year of age (age of den-independence). For interbirth interval, Δrank was calculated as the difference in rank between the birth of the first litter and the birth of the second litter. For annual reproductive success, Δrank was calculated as the difference between rank at the start and the end of the year of tenure. |
|  | Maternal rank | Integer or continuous variable representing the social rank of the mother on day of litter birth. In rare cases of adoption, we used the rank of the social mother, not the genetic mother [6]. When used as a covariate (in addition to the focal individual’s own rank), we use ordinal maternal rank only, as this better predicted cub survival (see results). |
|  | Maternal age | Continuous variable representing the age of mother in years on the day of litter birth. |
|  | Age | Continuous variable representing the age of focal individual in years on day of litter birth. |
|  | Litter status | Categorical variable representing litter size and within-litter dominance rank. Cubs with no siblings are "Singletons", while twins are "Dominant" or "Subordinate" depending on their within-litter dominance relationship. |
|  | Number of lactating females | Integer variable representing the number of females in the clan with cubs < 12 months of age ([3]) on the date of birth of the focal individual (for analyses of female age at first reproduction) or of their litter (for analyses of cub survival). |
|  | Sex | Categorical variable representing sex (“Male” or “Female”) of cub. |
|  | Litter size | Categorical variable (“1” or “2”) representing the size of the focal litter. Triplet litters are rare (1.2%) and were excluded from the analysis. |
|  | Male origin | Categorical variable (“Philopatric” or “Immigrant”) representing whether a male is sexually active in his birth clan or in a different clan. |
|  | Year of tenure | Categorical variable representing the number of years a male has been reproductively active in the current clan. Levels were “1”-“6” and an additional category for “7+”. These levels were used to mimic past analyses [7]. |
|  | Number of young females | Integer variable representing the number of females aged 1-5 years in the chosen breeding clan by a focal male. The number of young females at clan choice corresponds to the pool of females who are likely to choose an incoming male as sire based on known female preferences [8]. |
|  | Clan | Categorical variable representing one of the four or eight clans that the focal individual was a member of at age of first reproduction. |
| *Random* | ID | Categorical variable representing the identity of the focal individual to account for non-independence of repeated measures of the same individual. |
|  | Mother ID | Categorical variable representing the identity of the focal individual's mother to account for non-independence of the early life environment shared among siblings. |
|  | Year | Categorical variable representing the year of birth of a focal individual, used to control for any other differences in early life environment that vary annually. |

Table S2. Number and characteristics of outliers/sparse data and their justification for removal from each dataset used

| Trait dataset | Number of removed data points | Justification |
| --- | --- | --- |
| Interbirth interval | 7 | IBIs calculated < 9 months with at least one cub surviving. Biologically unlikely given gestation and lactation period. |
|  | 2 | IBIs calculated > 46 months. Likely due to “missing” litters. |
| Female AFR | 2 | AFR > 8 years. Likely to be due to “missing” an earlier litter. |
|  | 8 | Ordinal rank > 40. Data too sparse at low ranks leading to diagnostic issues. |
| Rank of mated female | 7 | Ordinal rank > 22. Data too sparse at low ranks leading to diagnostic issues. |
| Male AFR | 1 | AFR > 130 months. Only one point above 130 months at 163 months, leading to diagnostic issues. |
|  | 1 | Ordinal rank > 45. Only one point above ordinal rank 45 at ordinal rank 67, leading to diagnostic issues. |

# **S2. Supplementary result tables: model outputs**

Table S3. Model output for the probability of cub survival to 12 months for a focal female as a function of her ordinal rank, change in ordinal rank over 12 months, the twin status of the cub, sex of the cub, her age and the number of lactating females in the clan during this 12 month period. Shown are the variables by name, regression coefficients and standard errors (SE) on the logit scale, z values and their corresponding p values for each variable. Coefficients were estimated by generalised linear mixed model with a binomial distribution and a logit link function. This included a random effect on the intercept (female identity: variance = 0.21, year: variance = 17). The reference level for categorical variables were twin status “Dominant” and sex “Female”.

| Variable | Coefficient | SE | z value | *p* |
| --- | --- | --- | --- | --- |
| Intercept | 1.69 | 0.30 | 5.70 | < 0.01 |
| Ordinal rank | 0.02 | 0.01 | 3.74 | < 0.01 |
| ΔOrdinal rank | 0.02 | 0.02 | 1.54 | 0.12 |
| Twin status Singleton | -0.03 | 0.17 | -0.12 | 0.84 |
| Twin status Subordinate | -0.22 | 0.16 | -1.37 | 0.17 |
| Sex male | -0.24 | 0.14 | -1.74 | 0.08 |
| Age | 0.02 | 0.02 | 0.89 | 0.37 |
| Number of lactating females | -0.02 | 0.02 | -0.77 | 0.44 |

Table S4. Model output for the probability of cub survival to 12 months for a focal female as a function of her standardised rank, change in standardised rank over 12 months, the twin status of the cub, sex of the cub, her age and the number of lactating females in the clan during this 12 month period. Shown are the variables by name, regression coefficients and standard errors (SE) on the logit scale, z values and their corresponding p values for each variable. Coefficients were estimated by generalised linear mixed model with a binomial distribution and a logit link function. This included a random effect on the intercept (female identity: variance = 0.23, year: variance = 0.22). The reference level for categorical variables were twin status “Dominant” and sex “Female”.

| Variable | Coefficient | SE | z value | *p* |
| --- | --- | --- | --- | --- |
| Intercept | 1.35 | 0.32 | 4.22 | < 0.01 |
| Standardised rank | 0.42 | 0.16 | 2.60 | < 0.01 |
| ΔStandardised rank | 0.57 | 0.55 | 1.03 | 0.30 |
| Twin status Singleton | -0.05 | 0.17 | -0.34 | 0.74 |
| Twin status Subordinate | -0.22 | 0.166 | -1.40 | 0.16 |
| Sex male | -0.22 | 0.14 | -1.63 | 0.10 |
| Age | 0.02 | 0.02 | 0.85 | 0.40 |
| Number of lactating females | -0.02 | 0.02 | -1.20 | 0.23 |

Table S5. Model output for the probability of cub survival to 12 months for a focal female as a function of the twin status of the cub, sex of the cub, her age and the number of lactating females in the clan during this 12 month period. Shown are the variables by name, regression coefficients and standard errors (SE) on the logit scale, z values and their corresponding p values for each variable. Coefficients were estimated by generalised linear mixed model with a binomial distribution and a logit link function. This included a random effect on the intercept (female identity: variance = 0.28, year: variance = 0.21). The reference level for categorical variables were twin status “Dominant” and sex “Female”.

| Variable | Coefficient | SE | z value | *p* |
| --- | --- | --- | --- | --- |
| Intercept | 1.67 | 0.30 | 5.63 | < 0.01 |
| Twin status Singleton | -0.09 | 0.17 | -0.57 | 0.57 |
| Twin status Subordinate | -0.22 | 0.16 | -1.36 | 0.18 |
| Sex male | -0.24 | 0.14 | -1.72 | 0.09 |
| Age | 0.00 | 0.02 | 0.15 | 0.88 |
| Number of lactating females | -0.03 | 0.02 | -1.67 | 0.09 |

Table S6. Model output for the length of an interbirth interval in months for focal female as a function of her ordinal rank, a quadratic term of ordinal rank, change in ordinal rank over the interbirth interval, her age and litter size. Shown are the variables by name, regression coefficients and standard errors (SE) on an inverse square root transformation scale, z values and their corresponding p values for each variable. Coefficients were estimated by linear mixed model with a Gaussian distribution. This included a random effect on the intercept (female identity: variance < 0.01).

| Variable | Coefficient | Std. Error | z value | *p* |
| --- | --- | --- | --- | --- |
| Intercept | 0.26 | 6.52^-3^ | 37.77 | < 0.01 |
| Ordinal rank | 1.54e^-3^ | 2.72e^-4^ | 5.67 | < 0.01 |
| Ordinal rank (slope 2) | 1.37e^-5^ | 4.06e^-6^ | 3.36 | < 0.01 |
| ΔOrdinal rank | -2.40e^-4^ | 2.08e^-4^ | -1.16 | 0.28 |
| Age | -2.09e^-3^ | 4.74e^-4^ | -4.42 | < 0.01 |
| Litter size | 6.30e^-3^ | 2.56e^-3^ | 2.46 | 0.01 |

Table S7. Model output for the length of an interbirth interval in months for focal female as a function of her standardised rank, a quadratic term of standardised rank, change in standardised rank over the interbirth interval, her age and litter size. Shown are the variables by name, regression coefficients and standard errors (SE) on an inverse square root transformation scale, z values and their corresponding p values for each variable. Coefficients were estimated by linear mixed model with a Gaussian distribution. This included a random effect on the intercept (female identity: variance < 0.01).

| Variable | Coefficient | Std. Error | z value | *p* |
| --- | --- | --- | --- | --- |
| Intercept | 0.22 | 6.11e^-3^ | 36.77 | < 0.01 |
| Standardised rank | 0.01 | 4.36e^-3^ | 3.13 | < 0.01 |
| Standardised rank (slope 2) | 0.3 | 6.73e^-3^ | 3.81 | < 0.01 |
| ΔStandardised rank | 0.2 | 7.44e^-3^ | 2.17 | 0.03 |
| Age | -1.82e^-3^ | 4.68e^-4^ | -3.91 | < 0.01 |
| Litter size | 0.69e^-3^ | 2.50e^-3^ | 2.78 | < 0.01 |

Table S8. Model output for the length of an interbirth interval in months for focal female as a function of her age and litter size. Shown are the variables by name, regression coefficients and standard errors (SE) on an inverse square root transformation scale, z values and their corresponding p values for each variable. Coefficients were estimated by linear mixed model with a Gaussian distribution. This included a random effect on the intercept (female identity: variance < 0.01).

| Variable | Coefficient | Std. Error | z value | *p* |
| --- | --- | --- | --- | --- |
| Intercept | 0.24 | 6.12e^-3^ | 39.18 | < 0.01 |
| Age | -3.04e^-3^ | 4.74e^-4^ | -6.42 | < 0.01 |
| Litter size | 8.86e^-3^ | 8.86e^-3^ | 3.40 | < 0.01 |

Table S9. Model output for the age of first reproduction for a focal female as a function of her ordinal rank, a quadratic term of ordinal rank, change in ordinal rank over 12 months, the twin status of the female, her age and the number of lactating females in the clan during this 12 month period. Shown are the variables by name, regression coefficients and standard errors (SE) on a log-transformed scale, z values and their corresponding p values for each variable Coefficients were estimated by linear mixed model with a Gaussian distribution. This included a random effect on the intercept (identity of the female’s mother: variance < 0.01). The reference level for twin status was “Dominant”.

| Variable | Coefficient | Std. Error | z value | *p* |
| --- | --- | --- | --- | --- |
| Intercept | 3.58 | 0.05 | 72.52 | < 0.01 |
| Ordinal rank | -0.03 | 0.005 | -6.40 | < 0.01 |
| Ordinal rank (slope 2) | 4.52e^-4^ | 1.33e^-4^ | -3.41 | < 0.01 |
| ΔOrdinal rank | 0.02 | 0.004 | 5.23 | < 0.01 |
| Twin status Singleton | -0.03 | 0.30 | -1.03 | 0.30 |
| Twin status Subordinate | 0.01 | 0.29 | 0.40 | 0.69 |
| Age | -0.004 | 0.004 | -0.94 | 0.35 |
| Number of lactating females | -0.002 | 0.003 | -0.70 | 0.48 |

Table S10. Model output for the age of first reproduction for a focal female as a function of her standardised rank, a quadratic term for standardised rank, change in standardised rank over 12 months, the twin status of the female, her age and the number of lactating females in the clan during this 12 month period. Shown are the variables by name, regression coefficients and standard errors (SE) on a log-transformed scale, z values and their corresponding p values for each variable Coefficients were estimated by linear mixed model with a Gaussian distribution. This included a random effect on the intercept (identity of the female’s mother: variance < 0.01). The reference level for twin status was “Dominant”.

| Variable | Coefficient | Std. Error | z value | *p* |
| --- | --- | --- | --- | --- |
| Intercept | 3.95 | 0.05 | 74.98 | < 0.01 |
| Standardised rank | -0.16 | 0.06 | -2.81 | < 0.01 |
| Standardised rank (slope 2) | -0.32 | 0.08 | -4.17 | < 0.01 |
| ΔStandardised rank | -0.41 | 0.09 | -4.63 | < 0.01 |
| Twin status Singleton | -0.04 | 0.03 | -1.43 | 0.15 |
| Twin status Subordinate | 0.03 | 0.03 | 1.03 | 0.30 |
| Age | -0.005 | 0.005 | -1.05 | 0.29 |
| Number of lactating females | 0.01 | 0.003 | 3.16 | < 0.01 |

Table S11. Model output for the age of first reproduction for a focal female as a function of the twin status of the female, her age and the number of lactating females in the clan during this 12 month period. Shown are the variables by name, regression coefficients and standard errors (SE) on a log-transformed scale, z values and their corresponding p values for each variable Coefficients were estimated by linear mixed model with a Gaussian distribution. This included a random effect on the intercept (identity of the female’s mother: variance < 0.01). The reference level for twin status was “Dominant”.

| Variable | Coefficient | Std. Error | z value | *p* |
| --- | --- | --- | --- | --- |
| Intercept | 3.72 | 0.06 | 66.72 | < 0.01 |
| Twin status Singleton | -0.005 | 0.03 | -0.16 | 0.87 |
| Twin status Subordinate | 0.03 | 0.31 | 0.92 | 0.36 |
| Age | 0.006 | 0.005 | 1.15 | 0.25 |
| Number of lactating females | 0.007 | 0.004 | 1.81 | 0.07 |

Table S12. Model output for the annual reproductive success (number of cubs sired) of a focal male as a function of his ordinal rank, change in ordinal rank over 12 months, number of young females at time of clan choice, male origin (philopatric or immigrant), his maternal rank and an interaction term between male origin and tenure. Shown are the variables by name, regression coefficients and standard errors (SE) in log units, z values and their corresponding p values for each variable Coefficients were estimated by generalised linear mixed model with a negative binomial (nbinom2) distribution. This included a random effect on the intercept (identity of the focal male: variance = 0.25). The reference level for categorical variables were philopatric “FALSE” (immigrant) and tenure “1”.

| Variable | Coefficient | Std. Error | z value | *p* |
| --- | --- | --- | --- | --- |
| Intercept | -0.25 | 0.26 | -0.96 | 0.34 |
| Ordinal rank | 0.07 | 0.02 | 4.48 | < 0.01 |
| ΔOrdinal rank | 0.009 | 0.03 | 0.27 | 0.79 |
| Number of young females | 0.03 | 0.01 | 3.14 | < 0.01 |
| Philopatric TRUE | -1.21 | 0.41 | -2.94 | < 0.01 |
| Maternal rank | -3.78e^-4^ | 0.006 | -0.06 | 0.95 |
| Tenure 2 | 0.35 | 0.19 | 1.83 | 0.07 |
| Tenure 3 | 0.48 | 0.2 | 2.4 | 0.02 |
| Tenure 4 | 0.68 | 0.21 | 3.3 | < 0.01 |
| Tenure 5 | 0.34 | 0.23 | 1.5 | 0.13 |
| Tenure 6 | 0.15 | 0.25 | 0.61 | 0.54 |
| Tenure 7+ | -0.18 | 0.23 | -0.77 | 0.44 |
| Philopatric:Tenure 2 | -0.007 | 0.48 | -0.01 | 0.99 |
| Philopatric:Tenure 3 | 0.06 | 0.48 | 0.13 | 0.89 |
| Philopatric:Tenure 4 | -0.15 | 0.52 | -0.28 | 0.78 |
| Philopatric:Tenure 5 | 0.45 | 0.56 | 0.79 | 0.43 |
| Philopatric:Tenure 6 | 0.54 | 0.65 | 0.83 | 0.4 |
| Philopatric:Tenure 7+ | 0.23 | 0.59 | 0.39 | 0.69 |

Table S13. Model output for the annual reproductive success (number of cubs sired) of a focal male as a function of his standardised rank, change in standardised rank over 12 months, number of young females at time of clan choice, male origin (philopatric or immigrant) his maternal rank, tenure in the clan and an interaction term between male origin and tenure. Shown are the variables by name, regression coefficients and standard errors (SE) in log units, z values and their corresponding p values for each variable Coefficients were estimated by generalised linear mixed model with a negative binomial (nbinom2) distribution. This included a random effect on the intercept (identity of the focal male: variance = 0.29). The reference level for categorical variables were philopatric “FALSE” (immigrant) and tenure “1”.

| Variable | Coefficient | Std. Error | z value | *p* |
| --- | --- | --- | --- | --- |
| Intercept | -0.91 | 0.26 | -3.51 | < 0.01 |
| Standardised rank | 0.44 | 0.2 | 2.2 | 0.028 |
| Δ Standardised rank | 0.5 | 0.24 | 2.04 | 0.041 |
| Number of young females | 0.02 | 0.01 | 2.07 | 0.038 |
| Philopatric TRUE | -0.88 | 0.5 | -1.74 | 0.082 |
| Maternal rank | -9.18e^-4^ | 0.007 | -0.14 | 0.89 |
| Tenure 2 | 0.41 | 0.2 | 2.03 | 0.043 |
| Tenure 3 | 0.53 | 0.22 | 2.37 | 0.018 |
| Tenure 4 | 0.73 | 0.25 | 2.96 | < 0.01 |
| Tenure 5 | 0.4 | 0.28 | 1.45 | 0.15 |
| Tenure 6 | 0.23 | 0.31 | 0.73 | 0.46 |
| Tenure 7+ | -0.05 | 0.31 | -0.16 | 0.87 |
| Philopatric:Tenure 2 | -0.06 | 0.49 | -0.12 | 0.91 |
| Philopatric:Tenure 3 | 0.03 | 0.5 | 0.06 | 0.95 |
| Philopatric:Tenure 4 | -0.22 | 0.55 | -0.41 | 0.68 |
| Philopatric:Tenure 5 | 0.39 | 0.6 | 0.64 | 0.52 |
| Philopatric:Tenure 6 | 0.44 | 0.69 | 0.64 | 0.52 |
| Philopatric:Tenure 7+ | 0.11 | 0.63 | 0.17 | 0.86 |

Table S14. Model output for the annual reproductive success (number of cubs sired) of a focal male as a function of the number of young females at time of clan choice, male origin (philopatric or immigrant) his maternal rank, tenure in the clan and an interaction term between male origin and tenure. Shown are the variables by name, regression coefficients and standard errors (SE) in log units, z values and their corresponding p values for each variable Coefficients were estimated by generalised linear mixed model with a negative binomial (nbinom2) distribution. This included a random effect on the intercept (identity of the focal male: variance = 0.30). The reference level for categorical variables were philopatric “FALSE” (immigrant) and tenure “1”.

| Variable | Coefficient | Std. Error | z value | *p* |
| --- | --- | --- | --- | --- |
| Intercept | -1.03 | 0.22 | -4.7 | < 0.01 |
| Number of young females | 0.012 | 0.01 | 1.28 | 0.2 |
| Philopatric TRUE | -0.24 | 0.37 | -0.65 | 0.52 |
| Maternal rank | -9.66e^-4^ | 0.007 | -0.14 | 0.89 |
| Tenure 2 | 0.5 | 0.19 | 2.61 | < 0.01 |
| Tenure 3 | 0.74 | 0.2 | 3.78 | < 0.01 |
| Tenure 4 | 0.99 | 0.2 | 4.93 | < 0.01 |
| Tenure 5 | 0.72 | 0.22 | 3.33 | < 0.01 |
| Tenure 6 | 0.58 | 0.24 | 2.42 | 0.016 |
| Tenure 7+ | 0.39 | 0.2 | 1.95 | 0.051 |
| Philopatric:Tenure 2 | -0.17 | 0.48 | -0.36 | 0.72 |
| Philopatric:Tenure 3 | -0.22 | 0.48 | -0.46 | 0.64 |
| Philopatric:Tenure 4 | -0.53 | 0.52 | -1.01 | 0.31 |
| Philopatric:Tenure 5 | -0.02 | 0.56 | -0.04 | 0.97 |
| Philopatric:Tenure 6 | 0.004 | 0.65 | 0.006 | 0.996 |
| Philopatric:Tenure 7+ | -0.34 | 0.59 | -0.58 | 0.56 |

Table S15. Model output for the rank of a female with whom a focal male successfully sired cubs with as a function of his ordinal rank, the number of young females at time of clan choice, male origin (philopatric or immigrant) his maternal rank, tenure in the clan and an interaction term between male origin and tenure. Shown are the variables by name, regression coefficients and standard errors (SE) on a square root-transformed scale, z values and their corresponding p values for each variable Coefficients were estimated by linear mixed model with a Gaussian distribution. This included a random effect on the intercept (identity of the focal male: variance = 0.23). The reference level for categorical variables were philopatric “FALSE” (immigrant) and tenure “1”.

| Variable | Coefficient | Std. Error | z value | *p* |
| --- | --- | --- | --- | --- |
| Intercept | 2.33 | 0.34 | 6.83 | < 0.01 |
| Ordinal rank | -0.11 | 0.021 | -5.13 | < 0.01 |
| Number of young females | 0.03 | 0.01 | 2.44 | 0.015 |
| Philopatric TRUE | 0.34 | 0.59 | 0.57 | 0.57 |
| Maternal rank | -0.009 | 0.009 | -1.02 | 0.31 |
| Tenure 2 | 0.28 | 0.28 | 1 | 0.32 |
| Tenure 3 | 0.2 | 0.28 | 0.72 | 0.47 |
| Tenure 4 | 0.42 | 0.28 | 1.5 | 0.13 |
| Tenure 5 | 0.48 | 0.31 | 1.56 | 0.12 |
| Tenure 6 | 0.43 | 0.34 | 1.25 | 0.21 |
| Tenure 7+ | 0.3 | 0.32 | 0.96 | 0.34 |
| Philopatric:Tenure 2 | -1.3 | 0.73 | -1.8 | 0.072 |
| Philopatric:Tenure 3 | -0.63 | 0.72 | -0.88 | 0.38 |
| Philopatric:Tenure 4 | -0.82 | 0.74 | -1.11 | 0.27 |
| Philopatric:Tenure 5 | -0.31 | 0.84 | -0.37 | 0.71 |
| Philopatric:Tenure 6 | -0.46 | 0.97 | -0.48 | 0.63 |
| Philopatric:Tenure 7+ | -0.18 | 0.9 | -0.2 | 0.84 |

Table S16. Model output for the rank of a female with whom a focal male successfully sired cubs with as a function of his standardised rank, the number of young females at time of clan choice, male origin (philopatric or immigrant) his maternal rank, tenure in the clan and an interaction term between male origin and tenure. Shown are the variables by name, regression coefficients and standard errors (SE) on a square root-transformed scale, z values and their corresponding p values for each variable Coefficients were estimated by linear mixed model with a Gaussian distribution. This included a random effect on the intercept (identity of the focal male: variance = 0.23). The reference level for categorical variables were philopatric “FALSE” (immigrant) and tenure “1”.

| Variable | Coefficient | Std. Error | z value | *p* |
| --- | --- | --- | --- | --- |
| Intercept | 2.95 | 0.31 | 9.45 | < 0.01 |
| Standardised rank | -0.7 | 0.23 | -2.98 | < 0.01 |
| Number of young females | 0.04 | 0.01 | 3.17 | < 0.01 |
| Philopatric TRUE | 0.27 | 0.68 | 0.4 | 0.69 |
| Maternal rank | -0.005 | 0.009 | -0.56 | 0.57 |
| Tenure 2 | 0.35 | 0.29 | 1.19 | 0.23 |
| Tenure 3 | 0.32 | 0.31 | 1.04 | 0.3 |
| Tenure 4 | 0.6 | 0.31 | 1.92 | 0.06 |
| Tenure 5 | 0.65 | 0.35 | 1.86 | 0.06 |
| Tenure 6 | 0.64 | 0.39 | 1.64 | 0.1 |
| Tenure 7+ | 0.41 | 0.39 | 1.06 | 0.29 |
| Philopatric:Tenure 2 | -1.31 | 0.74 | -1.76 | 0.08 |
| Philopatric:Tenure 3 | -0.77 | 0.75 | -1.04 | 0.3 |
| Philopatric:Tenure 4 | -0.98 | 0.77 | -1.27 | 0.2 |
| Philopatric:Tenure 5 | -0.52 | 0.89 | -0.58 | 0.56 |
| Philopatric:Tenure 6 | -0.69 | 1.01 | -0.68 | 0.5 |
| Philopatric:Tenure 7+ | -0.33 | 0.94 | -0.35 | 0.72 |

Table S17. Model output for the rank of a female with whom a focal male successfully sired cubs with as a function of the number of young females at time of clan choice, male origin (philopatric or immigrant) his maternal rank, tenure in the clan and an interaction term between male origin and tenure. Shown are the variables by name, regression coefficients and standard errors (SE) on a square root-transformed scale, z values and their corresponding p values for each variable Coefficients were estimated by linear mixed model with a Gaussian distribution. This included a random effect on the intercept (identity of the focal male: variance = 0.32). The reference level for categorical variables were philopatric “FALSE” (immigrant) and tenure “1”.

| Variable | Coefficient | Std. Error | z value | *p* |
| --- | --- | --- | --- | --- |
| Intercept | 3.23 | 0.31 | 10.49 | < 0.01 |
| Number of young females | 0.05 | 0.013 | 4.11 | < 0.01 |
| Philopatric TRUE | -0.94 | 0.56 | -1.68 | 0.09 |
| Maternal rank | -0.004 | 0.009 | -0.47 | 0.64 |
| Tenure 2 | 0.18 | 0.29 | 0.64 | 0.52 |
| Tenure 3 | -0.05 | 0.28 | -0.16 | 0.87 |
| Tenure 4 | 0.19 | 0.28 | 0.67 | 0.51 |
| Tenure 5 | 0.13 | 0.31 | 0.42 | 0.67 |
| Tenure 6 | 0.07 | 0.34 | 0.21 | 0.83 |
| Tenure 7+ | -0.34 | 0.30 | -1.12 | 0.26 |
| Philopatric:Tenure 2 | -0.91 | 0.74 | -1.23 | 0.22 |
| Philopatric:Tenure 3 | -0.24 | 0.73 | -0.33 | 0.74 |
| Philopatric:Tenure 4 | -0.37 | 0.75 | -0.49 | 0.62 |
| Philopatric:Tenure 5 | 0.43 | 0.84 | 0.51 | 0.61 |
| Philopatric:Tenure 6 | 0.15 | 0.98 | 0.15 | 0.88 |
| Philopatric:Tenure 7+ | 0.53 | 0.92 | 0.58 | 0.56 |

Table S18. Model output for the age of first reproduction for a focal male as a function of his maternal ordinal rank at birth, change in ordinal rank (over 12 months), the number of young females at time of clan choice, his twin status, and the identity of the clan he was member to when he first reproduced. Shown are the variables by name, regression coefficients and standard errors (SE) on a log-transformed scale, z values and their corresponding p values for each variable Coefficients were estimated by linear model with a Gaussian distribution. The reference level for categorical variables were twin status “Dominant” and clan “Airstrip”.

| Variable | Coefficient | Std. Error | z value | *p* |
| --- | --- | --- | --- | --- |
| Intercept | 4.02 | 0.09 | 46.37 | < 0.01 |
| Ordinal rank | -0.004 | 0.002 | -1.72 | 0.09 |
| ΔOrdinal rank | 0.005 | 0.007 | 0.71 | 0.48 |
| Number of young females | 8.68e^-4^ | 0.003 | 0.25 | 0.8 |
| Twin status Singleton | 0.09 | 0.06 | 1.5 | 0.13 |
| Twin status Subordinate | 0.02 | 0.05 | 0.37 | 0.71 |
| Clan Lemala | -0.05 | 0.07 | -0.74 | 0.46 |
| Clan Munge | -0.01 | 0.07 | -0.17 | 0.86 |
| Clan Shamba | -0.004 | 0.06 | -0.06 | 0.95 |

Table S19. Model output for the age of first reproduction for a focal male as a function of his maternal standardised rank at birth, change in standardised rank (over 12 months), the number of young females at time of clan choice, his twin status, and the identity of the clan he was member to when he first reproduced. Shown are the variables by name, regression coefficients and standard errors (SE) on a log-transformed scale, z values and their corresponding p values for each variable Coefficients were estimated by linear model with a Gaussian distribution. The reference level for categorical variables were twin status “Dominant” and clan “Airstrip”.

| Variable | Coefficient | Std. Error | z value | *P* |
| --- | --- | --- | --- | --- |
| Intercept | 4.12 | 0.08 | 49.58 | < 0.01 |
| Standardised rank | -0.1 | 0.05 | -1.99 | 0.05 |
| ΔStandardised rank | 0.21 | 0.19 | 1.13 | 0.26 |
| Number of young females | 0.001 | 0.003 | 0.42 | 0.68 |
| Twin status Singleton | 0.08 | 0.06 | 1.31 | 0.19 |
| Twin status Subordinate | 0.017 | 0.05 | 0.32 | 0.75 |
| Clan Lemala | -0.07 | 0.07 | -1.06 | 0.29 |
| Clan Munge | -0.03 | 0.07 | -0.45 | 0.65 |
| Clan Shamba | 0.01 | 0.06 | 0.18 | 0.85 |

Table S20. Model output for the age of first reproduction for a focal male as a function of the number of young females at time of clan choice, his twin status, and the identity of the clan he was member to when he first reproduced. Shown are the variables by name, regression coefficients and standard errors (SE) on a log-transformed scale, z values and their corresponding p values for each variable Coefficients were estimated by linear model with a Gaussian distribution. The reference level for categorical variables were twin status “Dominant” and clan “Airstrip”.

| Variable | Coefficient | Std. Error | z value | *P* |
| --- | --- | --- | --- | --- |
| Intercept | 4.08 | 0.082 | 49.49 | < 0.01 |
| Number of young females | 4.57e^-4^ | 0.003 | 0.13 | 0.89 |
| Twin status Singleton | 0.09 | 0.06 | 1.57 | 0.12 |
| Twin status Subordinate | 0.02 | 0.05 | 0.33 | 0.74 |
| Clan Lemala | -0.06 | 0.07 | -0.86 | 0.39 |
| Clan Munge | -0.02 | 0.07 | -0.32 | 0.75 |
| Clan Shamba | 0.008 | 0.06 | 0.13 | 0.9 |

# **S3. Supplementary results: constant covariate values for marginal effects**

To visualise the effect of ordinal or standardised rank on the six reproductive traits, all other covariates were held constant. Marginal effects were obtained using the *ggpredict()* function of R package *ggeffects* version 1.6.0 [9]. Below we detail the values at which covariates were held constant: numeric variables are set to the mean and factors to their reference level. Although we modelled tenure as a factor, we manually set the value to hold it constant at as “3”. Relatively few males sire cubs in their first years of tenure. Males in our study population are most successful in years 3-4 of tenure (for immigrants, 3+ for philopatrics) [5], and so this better represents the population average than using tenure “1”. As models for each reproductive trait are identical with the exception of alternative rank-related terms (e.g., Δrank), we provide the constant values for each dataset only once to avoid repetition.

*Female: cub survival*

ΔOrdinal rank = 2; ΔStandardised rank = -0.04; twin status = “Dominant”; sex = “Female”; age = 6.93; number of lactating females = 10.

*Female: interbirth interval*

ΔOrdinal rank = -2; ΔStandardised rank = -0.06; age = 8.27; litter size = 2.

*Female: age at first reproduction*

ΔOrdinal rank = 1; ΔStandardised rank = -0.03; twin status = “Dominant”; age = 6.81; number of lactating females = 9.

*Male: annual reproductive success*

ΔOrdinal rank =-1, ΔStandardised rank = 0.15, number of young females = 15, philopatric = “FALSE”, maternal rank = 9, tenure = “3”.

*Male: mate rank*

Number of young females = 16, philopatric = “FALSE”, maternal rank = 9, tenure = “3”.

*Male: age at first reproduction*

ΔOrdinal rank = 1, ΔStandardised rank = -0.02, Number of young females = 15, twin status = “Dominant”, clan = “Airstrip”.

# **Supplementary references**

1. Altmann J. 2001 *Baboon Mothers and Infants*. University of Chicago Press.

2. Matthews LH. 1939 Reproduction in the spotted hyaena, Crocuta crocuta (erxleben). *Philosophical Transactions of the Royal Society of London. Series B, Biological Sciences* **230**, 1–78. (doi:10.1098/rstb.1939.0004)

3. Gicquel M, East ML, Hofer H, Benhaiem S. 2022 Early‐life adversity predicts performance and fitness in a wild social carnivore. *Journal of Animal Ecology* **91**, 2074–2086. (doi:10.1111/1365-2656.13785)

4. Höner OP, Wachter B, Hofer H, Wilhelm K, Thierer D, Trillmich F, Burke T, East ML. 2010 The fitness of dispersing spotted hyaena sons is influenced by maternal social status. *Nature Communications* **1**, 1–7. (doi:10.1038/ncomms1059)

5. Davidian E, Courtiol A, Wachter B, Hofer H, Honer OP. 2016 Why do some males choose to breed at home when most other males disperse? *Science Advances* **2**. (doi:10.1126/sciadv.1501236)

6. East ML, Honer OP, Wachter B, Wilhelm K, Burke T, Hofer H. 2009 Maternal effects on offspring social status in spotted hyenas. *Behav. Ecol.* **20**, 478–483. (doi:10.1093/beheco/arp020)

7. Davidian E, Courtiol A, Wachter B, Hofer H, Höner OP. 2016 Why do some males choose to breed at home when most other males disperse? *Science Advances* **2**, e1501236. (doi:10.1126/sciadv.1501236)

8. Höner OP, Wachter B, East ML, Streich WJ, Wilhelm K, Burke T, Hofer H. 2007 Female mate-choice drives the evolution of male-biased dispersal in a social mammal. *Nature* **448**, 798–801. (doi:10.1038/nature06040)

9. Lüdecke D. 2018 ggeffects: Tidy Data Frames of Marginal Effects from Regression Models. *Journal of Open Source Software* **3**, 772. (doi:10.21105/joss.00772)
